# A Review of *Treatment and Management of Tropical Liver Disease* by Jose Debes

**DOI:** 10.4269/ajtmh.24-0383

**Published:** 2024-08-27

**Authors:** Peyton Thompson

**Affiliations:** Department of Pediatrics, Division of Infectious Diseases University of North Carolina at Chapel Hill Chapel Hill, North Carolina E-mail: peyton_thompson@med.unc.edu

This first edition of *Treatment and Management of Tropical Liver Disease* is a timely addition to the tropical medicine literature. It is an impressively comprehensive and concise guide to both infectious and non-infectious causes of liver disease that affect people worldwide, and especially in tropical/subtropical regions. It is portable and compact, an easy read at just over 270 pages. The five well-organized sections range from viral hepatitides, liver masses, tropical infectious and non-infectious diseases, to rare tropical diseases that affect the liver. Each of the 35 chapters includes an overview and sections on epidemiology, pathology/pathogenesis, clinical presentation, diagnosis, and treatment/management. Key points highlight relevant takeaways from each chapter. Editor Jose Debes outperformed himself in masterfully assembling this handy clinical reference.

Of particular interest and importance is the emphasis on epidemiology and impacts of the various liver diseases on people living in tropical regions. The authors, who are experts in their fields from across the globe, adeptly integrate maps of high-prevalence areas combined with up-to-date literature and descriptions of clinical presentations by region of the world. Furthermore, authors provide context for clinical decision-making based on local availability, or lack thereof, of resources. This allows for clinicians in all settings to make use of this reference. It was most fascinating to learn of the cultural practices associated with the acquisition of various diseases. For instance, Chapter 12–*Opisthorchis* and *Clonorchis* provides an in-depth description of “raw attitudes,” the ingrained cultural practice of ingestion of raw food, and in particular raw fish, which is associated with infection by liver flukes. Another eye-opening example is the association of iron overload in sub-Saharan Africa with traditional home-brewed beer, prepared in iron or steel containers (illustrated in Figure 35.3) that leach over 40 mg/L of iron. Without understanding the cultural context of these diseases, prevention and management would be nearly impossible.

Most chapters include unique aspects of their respective topics, such as life cycles of parasites and differential diagnoses and historical accounts of pathogens. Regarding the latter, the historical overview section in Chapter 32–The Liver in Q Fever provides a concise but intriguing history of the discovery of *Coxiella burnetii,* the causative agent of Q fever. Interestingly, *C. burnetii* was once named *Rickettsia burnetii* but moved in 1938 into its own genus of *Coxsiella*, as it was considered a proteobacterium rather than part of the Rickettsiae family. The tables and figures are helpful quick references. For instance, Table 26.1–Screening and Confirmatory Testing for Etiology of Acute Liver Failure, walks the reader through first- and second-tier evaluations for a range of causes of liver failure, whereas Figure 26.2–Suggested Flow Chart for Evaluation of Liver Injury, just below, takes the provider step-by-step through a workup ending with liver biopsy, if available and/or necessary. An inclusive differential diagnosis of hepatic granulomas in the chapter on hepatic tuberculosis is included in Table 13.3–Causes of Hepatic Granulomas. Differential diagnoses such as these can be useful to a range of trainees and clinicians across the globe.

In summary, *Treatment and Management of Tropical Liver Disease* is a highly useful, easy-to-read, and comprehensive guide to infectious and non-infectious causes of liver disease in tropical (and non-tropical) areas of the world. I would recommend this as a reference for all medical trainees and personnel living in and traveling to the tropics, whether they routinely or rarely provide care for patients with liver disease.[Bibr b1]
